# *Dj1* deficiency protects against atherosclerosis with anti-inflammatory response in macrophages

**DOI:** 10.1038/s41598-021-84063-6

**Published:** 2021-02-25

**Authors:** Tharini Sivasubramaniyam, Jiaqi Yang, Henry S. Cheng, Alexandra Zyla, Angela Li, Rickvinder Besla, Idit Dotan, Xavier S. Revelo, Sally Yu Shi, Helen Le, Stephanie A. Schroer, David W. Dodington, Yoo Jin Park, Min Jeong Kim, Daniella Febbraro, Isabelle Ruel, Jacques Genest, Raymond H. Kim, Tak W. Mak, Daniel A. Winer, Clinton S. Robbins, Minna Woo

**Affiliations:** 1 Toronto General Hospital Research Institute, University Health Network, Toronto, ON M5G 2C4 Canada; 2 Institute of Medical Science, University of Toronto, Toronto, ON M5G 2M9 Canada; 3 Department of Immunology, University of Toronto, Toronto, ON M5G 2M9 Canada; 4 Department of Laboratory Medicine and Pathobiology, University of Toronto, Toronto, ON M5G 2M9 Canada; 5 Institute of Medical Research, Kangbuk Samsung Hospital, Sungkyunkwan University School of Medicine, Seoul, 03181 Korea; 6 Research Institute of the McGill University Health Centre, Royal Victoria Hospital, Montreal, QC H4A 3J1 Canada; 7 Department of Medicine, McGill University, Royal Victoria Hospital, Montreal, QC H4A 3J1 Canada; 8 Department of Medicine, University Health Network/Sinai Health System, University of Toronto, Toronto, ON M5G 2C4 Canada; 9 Department of Pathology, University Health Network, Toronto, M5G 2C4 Canada; 10 Division of Endocrinology and Metabolism, University Health Network/Sinai Health System, University of Toronto, Toronto, ON M5G 2C4 Canada; 11 MaRS Centre, Toronto Medical Discovery Tower, 101 College Street, 10th floor, Room 10-361, Toronto, ON M5G 1L7 Canada

**Keywords:** Atherosclerosis, Mouse, Animal disease models, Mechanisms of disease

## Abstract

Inflammation is a key contributor to atherosclerosis with macrophages playing a pivotal role through the induction of oxidative stress and cytokine/chemokine secretion. DJ1, an anti-oxidant protein, has shown to paradoxically protect against chronic and acute inflammation. However, the role of DJ1 in atherosclerosis remains elusive. To assess the role of *Dj1* in atherogenesis, we generated whole-body *Dj1*-deficient atherosclerosis-prone *Apoe* null mice (Dj1^*−/−*^*Apoe*^*−/−*^). After 21 weeks of atherogenic diet, *Dj1*^*−/−*^* Apoe*^*−/−*^mice were protected against atherosclerosis with significantly reduced plaque macrophage content. To assess whether haematopoietic or parenchymal *Dj1* contributed to atheroprotection in *Dj1*-deficient mice, we performed bone-marrow (BM) transplantation and show that *Dj1*-deficient BM contributed to their attenuation in atherosclerosis. To assess cell-autonomous role of macrophage *Dj1* in atheroprotection, BM-derived macrophages from *Dj1*-deficient mice and *Dj1*-silenced macrophages were assessed in response to oxidized low-density lipoprotein (oxLDL). In both cases, there was an enhanced anti-inflammatory response which may have contributed to atheroprotection in *Dj1*-deficient mice. There was also an increased trend of plasma DJ-1 levels from individuals with ischemic heart disease compared to those without. Our findings indicate an atheropromoting role of *Dj1* and suggests that targeting *Dj1* may provide a novel therapeutic avenue for atherosclerosis treatment or prevention.

## Introduction

Cardiovascular diseases (CVD) stemming largely from atherosclerosis remain the leading cause of morbidity and mortality globally despite recent therapeutic advances. Atherosclerosis is a chronic inflammatory disease characterized by the narrowing of blood vessels by the growth of lipid-rich plaques. Overabundance of plasma lipids from genetic predisposition and cholesterol-rich diets elevates the circulatory levels of oxidized low-density lipoproteins (oxLDL) and promotes their deposition in vessel walls eliciting an inflammatory response. Circulating monocytes that are recruited and differentiated into macrophages are tasked with the clearance of excess cholesterol-rich lipids. Ineffective clearance leads to lipid-laden macrophages differentiating into pro-inflammatory foam cells. Upon their death, foam cells leave behind a fatty lipid streak within the vessel wall allowing for growth of atheromas. As such, defects in macrophage function have long been viewed as detrimental to cardiovascular health.

Metabolic abnormalities caused by excess fuel availability have been strongly associated with the prevalence of CVD. For example, CVD in individuals with obesity has worse clinical outcomes compared to those without. In particular, the overproduction of reactive oxygen species (ROS) as a by-product of cellular metabolism results in redox imbalance and oxidative stress. While oxidative stress from ROS have been considered a risk factor for the pathogenesis of atherosclerosis, emerging evidence highlights beneficial roles of ROS in metabolism, such as during exercise and caloric restriction^1^. Thus, potential paradoxical beneficial role of ROS in cardiovascular pathophysiology has stimulated investigation of antioxidant proteins as potential therapeutic interventions in atherosclerosis.

DJ-1 (PARK7) is a highly conserved antioxidant protein first discovered with mutations linked to a rare form of familial early-onset Parkinson’s disease^2^. Ablation of *Dj1* in mice increased oxidative stress to neurons resulting in susceptibility to cell death, whereas overexpression of *Dj1* provided protection^2,3^. In skeletal muscle, a critical tissue in fuel homeostasis, DJ-1 is induced in response to high fat diet (HFD)^4^. In keeping with the anti-oxidative role of DJ-1, muscle of *Dj1*-deficient mice showed elevated levels of ROS. Interestingly, this increased ROS in myocytes led to a metabolic reprogramming and induction of Warburg-like aerobic glycolysis, culminating in increased energy expenditure in skeletal muscle without detrimental effects of oxidative stress and protection against diet induced obesity and diabetes^4^. Furthermore, recent findings revealed *Dj1*-deficiency to facilitate efficient phagocytic bacterial clearance during sepsis in mice and humans through increased ROS^5^. Collectively, DJ-1 is involved in the regulation of oxidative stress and inflammation, both aspects well appreciated to contribute to the pathogenesis of atherosclerosis. Yet, DJ-1’s role in atherogenesis remains unknown. Here, we show that genetic deletion of *Dj1* in a mouse model of atherosclerosis provides protection against lipid and macrophage deposition in the intimal region of the aorta.

## Results

### *Dj1* deficiency provides protection against atherosclerosis in mice

**Figure 1:**
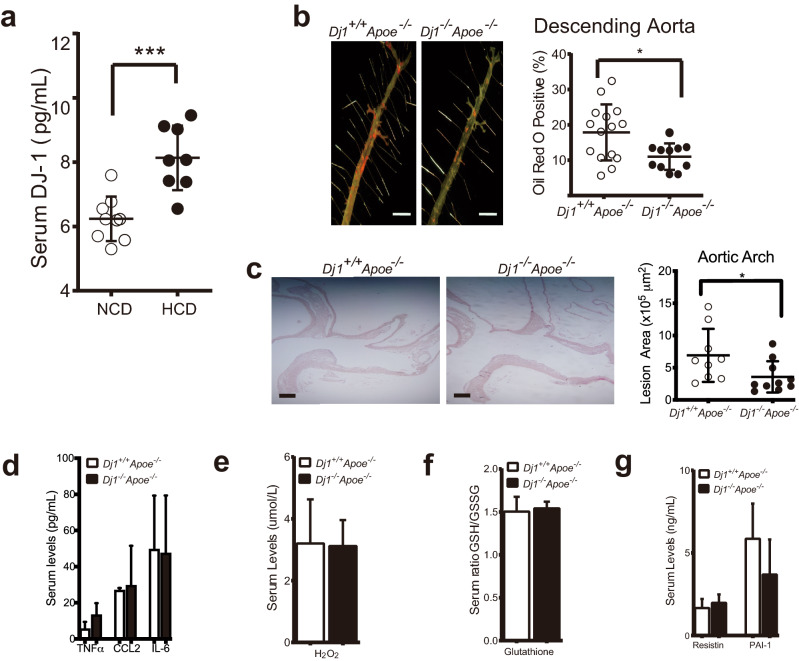
*Dj1*-deficiency provides protection against atherogenesis in mice. Serum collected from *Apoe*^*−/−*^ mice fed a normal chow or atherogenic diet for 16 weeks. (**a**) Serum levels of DJ-1 in normal chow diet (NCD)-fed *Apoe*^−/−^ (n = 9) and atherogenic high cholesterol diet (HCD)-fed *Apoe*^−/−^ (n = 8). Aortas and serum collected from mice after 21 weeks of atherogenic diet starting at 6 weeks. (**b**) Representative photographs of *en face* Oil-red-O (ORO) staining and quantification of atherosclerotic plaque area in descending aortas of *Dj1*^*−/−*^*Apoe*^*−/−*^ mice (n = 11) and control *Dj1*^+*/*+^*Apoe*^*−/−*^ mice (n = 15). Scale bar 5 mm. (**c**) Representative images of the lesser curvature of longitudinal aortic arch sections from male *Dj1*^*−/−*^*Apoe*^*−/−*^ mice (n = 10) and control *Dj1*^+*/*+^*Apoe*^*−/−*^ mice (n = 9) stained with H&E and quantification of lesion size at the lesser curvature. Scale bar 200 μm. (**d**) Serum levels of TNFα, CCL2 and IL-6 in *Dj1*^*−/−*^*Apoe*^*−/−*^ mice (n = 4–8) and control *Dj1*^+*/*+^*Apoe*^*−/−*^ mice (n = 2–7). (**e**) Serum levels of H_2_O_2_ in *Dj1*^*−/−*^*Apoe*^*−/−*^ mice (n = 9) and control *Dj1*^+*/*+^*Apoe*^*−/−*^ mice (n = 10). (**f**) Serum levels of glutathione in *Dj1*^*−/−*^*Apoe*^*−/−*^ mice (n = 7) and control *Dj1*^+*/*+^*Apoe*^*−/−*^ mice (n = 7). (**g**) Serum levels of Resistin and PAI-1 n *Dj1*^*−/−*^*Apoe*^*−/−*^ mice (n = 4) and control *Dj1*^+*/*+^*Apoe*^*−/−*^ mice (n = 4). *IL-6* interleukin 6, *TNFα* tumor necrosis factor α, *CCL2* C–C motif chemokine 2, *H*_*2*_*O*_*2*_ hydrogen peroxide, *PAI-1* plasminogen activator inhibitor-1. Data represent mean ± SD. Differences between groups were analyzed for statistical significance by Student unpaired t test and Wilcoxon Rank test. *P < 0.05, **P < 0.01.

Atherosclerosis-prone apolipoprotein E-null (*Apoe*^*−/−*^) mice placed on a high cholesterol high fat diet (HCD; 0.2% cholesterol; 60% kcal of fat) for 16 weeks showed a significant increase in serum Dj-1 levels compared to *Apoe*^*−/−*^ mice on normal chow diet (NCD) (0.02% cholesterol; 17% kcal of fat) (Fig. 1a). To elucidate the essential role of *Dj1* in atherogenesis, we generated global double knockout *Dj1*^*−/−*^*Apoe*^*−/−*^ mice by crossing *Dj1*^*−/−*^ mice with *Apoe*^*−/−*^ mice. These mice along with control *Dj1*^+*/*+^*Apoe*^*−/−*^littermates were placed on 21 weeks of HCD. Interestingly, male *Dj1*^*−/−*^*Apoe*^*−/−*^ accumulated less lipid-rich plaque burden in the descending aorta (Fig. 1b) and the aortic arch (Fig. 1c) compared to *Dj1*^+*/*+^*Apoe*^*−/−*^ mice. Females showed much milder disease in both genotypes with a trend to decreased plaque burden in both descending aorta and aortic arch (Supplementary Figure S1). Thus, for the remainder of the study, we focused on males for further characterization.

DJ-1 was recently shown to dampen inflammatory mediators with increased reactive oxygen species (ROS) during bacterial induced sepsis^5^. Inflammation and ROS are major mediators of atherogenesis. We therefore measured circulating levels of tumor necrosis factor alpha (TNFα), interleukin-6 (IL-6), C–C motif chemokine 2 (CCL2), and ROS such as hydrogen peroxide (H_2_O_2_) and glutathione from mice after 16 weeks of HCD. No significant differences were observed between the *Dj1*^+*/*+^*Apoe*^*−/−*^ and *Dj1*^*−/−*^* Apoe*^*−/−*^ mice (Fig. 1d–f). In addition, no differences of other circulating biomarkers of atherogenesis such as plasminogen activator inhibitor 1 (PAI-1) and the adipokine resistin were observed (Fig. 1g). Collectively, the lack of change to systemic atherogenic mediators suggests effect of *Dj1*-deficiency may be occurring locally within the plaque.

### *Dj1 *deficiency confers beneficial metabolic parameters during atherogenesis

**Figure 2:**
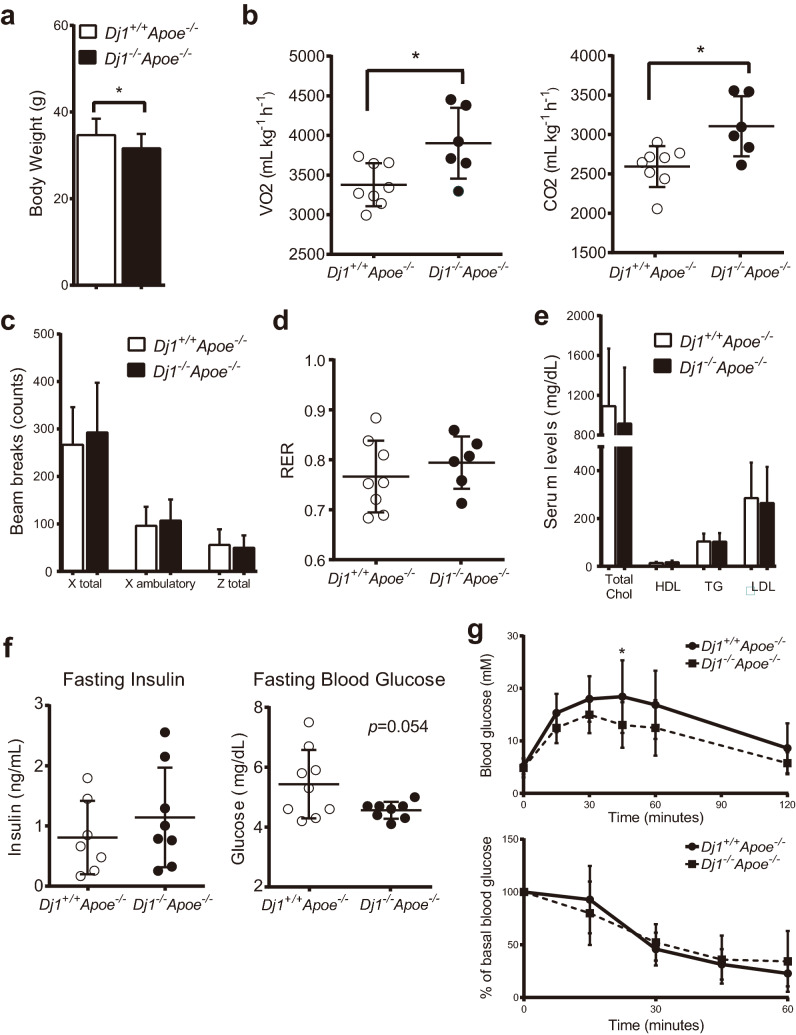
Metabolic parameters in *Dj1*-deficient *Apoe*-null mice. Results collected from mice after 21 weeks of atherogenic diet starting at 6 weeks. (**a**) Body weight of *Dj1*^*−/−*^*Apoe*^*−/−*^ mice (n = 13) and control *Dj1*^+*/*+^*Apoe*^*−/−*^ mice (n = 15). *Dj1*^*−/−*^*Apoe*^*−/−*^ mice (n = 6) and control *Dj1*^+*/*+^*Apoe*^*−/−*^ mice (n = 8) were housed individually in metabolic chambers with free access to food and water and energy balance data were collected for 24 h. Results are presented as (**b**) oxygen consumption (VO_2_) and carbon dioxide production (VCO_2_); (**c**) physical activity, expressed as average number of infra-red beam breaks during one measurement interval; (**d**) RER, calculated as VCO_2_/VO_2_. Total serum (**e**) cholesterol, HDL-cholesterol, LDL-cholesterol, and triglycerides in *Dj1*^*−/−*^*Apoe*^*−/−*^ mice (n = 7) and control *Dj1*^+*/*+^*Apoe*^*−/−*^ mice (n = 13). (**f**) Fasting serum insulin and blood glucose levels from *Dj1*^*–/–*^*Apoe*^*–/–*^ mice (*n* = 8) and control *Dj1*^+*/*+^*Apoe*^*–/–*^* mice* (*n* = 7–9). (**g**) Glucose tolerance test in overnight fasted *Dj1*^*−/−*^*Apoe*^*−/−*^ mice (n = 10) and control *Dj1*^+*/*+^*Apoe*^*−/−*^ mice (n = 14). Mice received glucose (1 g/kg) intraperitoneally and blood glucose was measured at 15, 30, 45, 60 and 120 min following injection. Insulin tolerance test in 4 h-fasted *Dj1*^*−/−*^*Apoe*^*−/−*^ mice (n = 9) and control *Dj1*^+*/*+^*Apoe*^*−/−*^ mice (n = 11). Mice received human regular insulin (0.5 units/kg) intraperitoneally and blood glucose was measured at 15, 30 and 45 min following injection. Data are expressed as a percentage of basal (fasting) glucose. Data represent mean ± SD. Differences between groups were analyzed for statistical significance by Student unpaired t test and multiple t test. *P < 0.05.

We have previously shown that *Dj1*-deficient mice are protected against diet-induced obesity and associated metabolic abnormalities such as insulin resistance and glucose intolerance^4^. Similarly, our *Dj1*^*−/−*^*Apoe*^*−/−*^ mice conferred less body weight gain after 21 weeks of HCD (Fig. 2a). These mice also demonstrated increased energy expenditure with elevated oxygen consumption and carbon dioxide exhaustion (Fig. 2b). This increased energy expenditure was not associated with any significant changes in physical activity (Fig. 2c) or fuel source utilization (Fig. 2d). Circulating lipid levels were not affected by *Dj1* deficiency as indicated by similar total-, high-density lipoprotein (HDL)-, and low-density lipoprotein (LDL)-cholesterol, and triglycerides (TG) between genotypes (Fig. 2e). In addition, fasting glucose and insulin levels, and glucose and insulin tolerance tests were not significantly altered between groups (Fig. 2f–g), suggesting that effects DJ-1 have on circulating lipids and glucose homeostasis may not play a major role in atherogenesis.

### Loss of *Dj1* leads to reduced macrophages within atherosclerotic plaques

**Figure 3:**
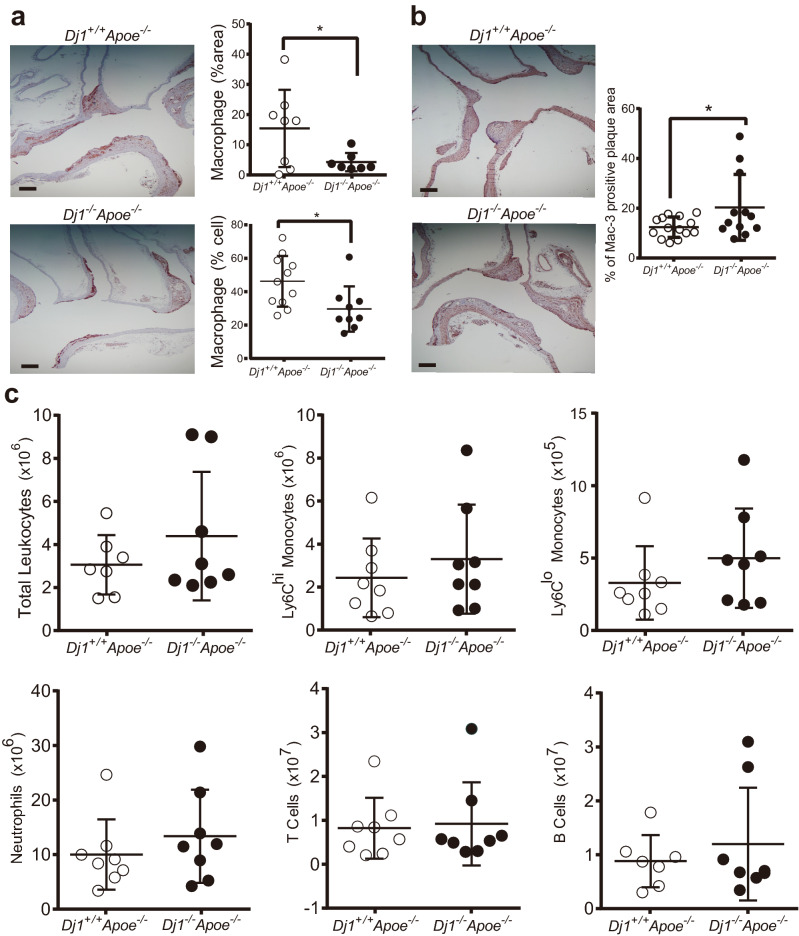
*Dj1*-deficiency leads to reduced macrophages within atherosclerotic plaques. Aortas and blood collected from mice after 21 weeks of atherogenic diet, starting at 6 weeks. (**a**) Lesser curvature of longitudinal aortic arch sections from *Dj1*^*−/−*^*Apoe*^*−/−*^ mice (n = 7–9) and control *Dj1*^+*/*+^*Apoe*^*−/−*^ mice (n = 8–11) immunostained for Mac-3 as well as quantification of positively stained area and cells as percentages. Scale bar 200 μm. (**b**) Representative images of the lesser curvature of longitudinal aortic arch sections from male *Dj1*^*−/−*^*Apoe*^*−/−*^ mice (n = 12) and control *Dj1*^+*/*+^*Apoe*^*−/−*^ mice (n = 14) stained with α-smooth muscle actin (α-SMA) and quantification of positively stained area as a percentage. Scale bar 200 μm. (**c**) Frequency and absolute numbers of Ly6C^hi^ monocytes, Ly6C^lo^ monocytes, neutrophils, T cells and B cells in red cell–lysed blood from *Dj1*^*–/–*^*Apoe*^*–/–*^ mice (*n* = 8) and control *Dj1*^+*/*+^*Apoe*^*–/–*^ mice (*n* = 7–8) using flow cytometry. Data represent mean ± SD. Differences between groups were analyzed for statistical significance by Student unpaired t test and Wilcoxon Rank test. *P < 0.05.

To further investigate the reduction of plaque size in the *Dj1*^*−/−*^*Apoe*^*−/−*^ mice, quantification of macrophages and smooth muscle cells (SMCs) by immunohistochemical analysis on sagittal sectioned aortic arches was performed. Interestingly, *Dj1*^*−/−*^*Apoe*^*−/−*^ atherosclerotic plaques harboured substantially less Mac3^+^ macrophages (Fig. 3a), while smooth muscle actin (SMA)^+^ SMCs were increased compared to *Dj1*^+*/*+^*Apoe*^*−/−*^ mice which can be in keeping with increased plaque stability in these mice (Fig. 3b). Furthermore, circulatory leukocytes, Ly6C^hi^ and Ly6C^lo^ monocytes, neutrophils, and B and T lymphocytes were quantified by flow cytometry revealing no significant differences (Fig. 3c). This suggests that the reduction of lesional macrophages is not due to a systemic effect but rather due to that localized within the plaque.

### Deletion of *Dj1* in bone marrow-derived cells leads to reduced atherosclerotic burden and plaque macrophages

**Figure 4:**
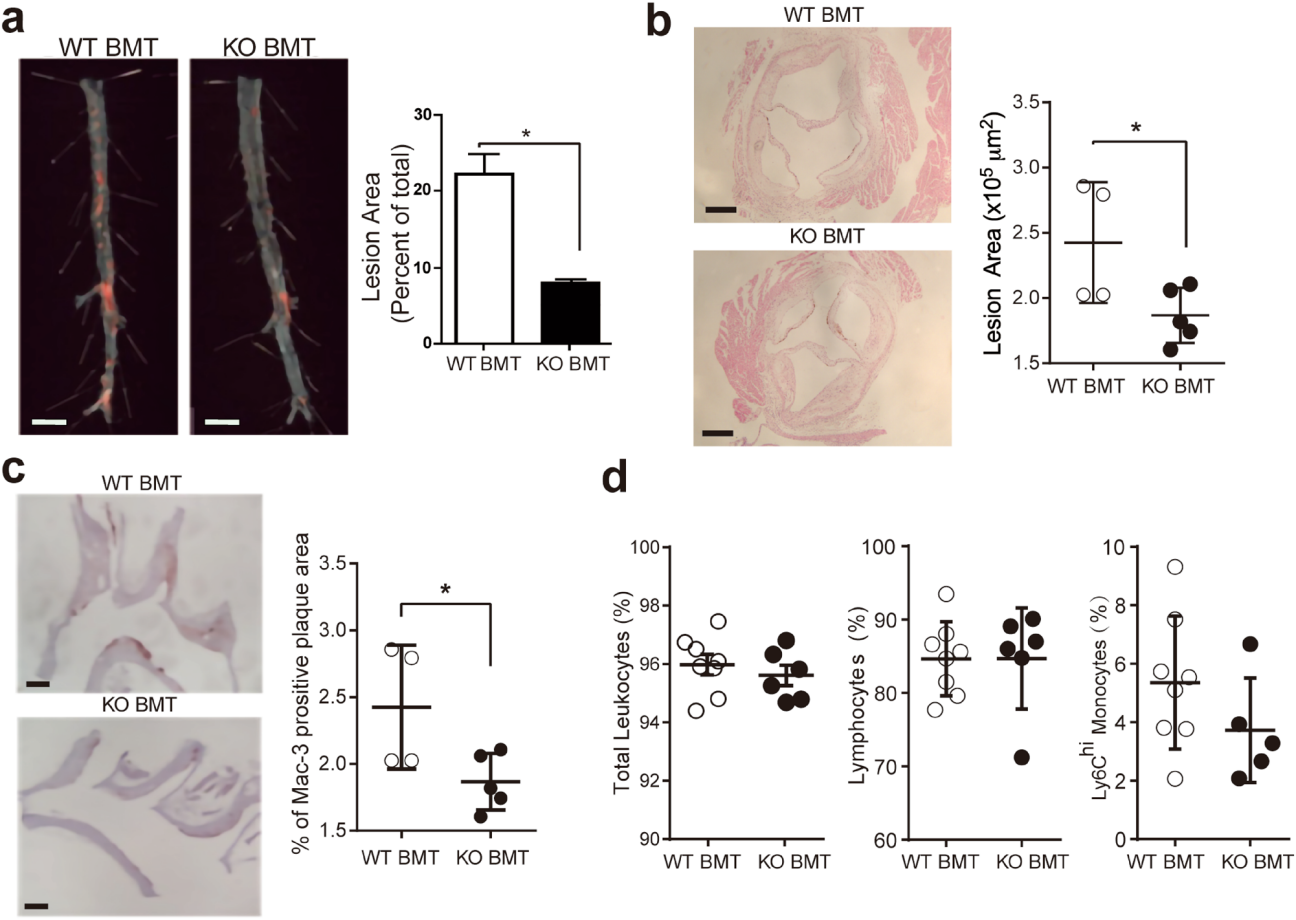
*Dj1* deletion in haematopoietic cells contribute to reduced atherosclerotic plaques and macrophages. Lethally irradiated *Apoe*^*−/−*^ mice given bone marrow transplantation (BMT) from wild-type *Dj1*^+*/*+^*Apoe*^*–/–*^ (WT BMT) donors or *Dj1*^*–/–*^*Apoe*^*–/–*^ mice (KO BMT) followed by atherogenic diet for 16 weeks. (**a**) Representative photographs of *en face* Oil-red-O (ORO) staining and quantification of atherosclerotic plaque area in descending aortas of KO BMT mice (n = 6) and WT BMT mice (n = 8). Scale bar 5 mm. (**b**) Representative images of the aortic root sections from KO BMT mice (n = 5) and WT BMT mice (n = 4) stained with H&E and quantification of lesion size. Scale bar 200 μm. (**c**) Representative images of the lesser curvature of longitudinal aortic arch sections from KO BMT mice (n = 6) and WT BMT mice (n = 8) stained with α-smooth muscle actin (α-SMA) and quantification of positively stained area as a percentage. Scale bar 200 μm. (**d**) Frequency of lymphocytes and Ly6C^hi^ monocytes in red cell-lysed blood from KO BMT mice (*n* = 5–6) and WT BMT mice (*n* = 8) using flow cytometry. Data represent mean ± SD. Differences between groups were analyzed for statistical significance by Student unpaired t test and Wilcoxon Rank test. *P < 0.05, **P < 0.01.

Next, we performed bone marrow transplantation (BMT) to elucidate the role of DJ-1 in hematopoietic cells during atherogenesis. *Apoe*^*−/−*^ mice were irradiated lethally and reconstituted with BM from either *Dj1*^+*/*+^*Apoe*^*−/−*^ or *Dj1*^*−/−*^*Apoe*^*−/−*^ mice (referred herein as WT or KO BMT respectively). After 8 weeks of recovery and reconstitution, these mice were placed on 16 weeks of HCD. Similar the global *Dj1*^*−/−*^*Apoe*^*−/−*^ mice, KO BMT mice also had reduced atherosclerotic plaque burden in the aorta and root (Fig. 4a,b). These plaques also comprised of less mac3^+^ macrophages (Fig. 4c). Circulating Ly6C^hi^ monocytes and lymphoid cells were similar between the groups, which mirrors the phenotype observed in the untransplanted *Dj1*^*−/−*^*Apoe*^*−/−*^ mice (Fig. 4d). Similar reduction of plaque macrophages between global *Dj1*^*−/−*^*Apoe*^*−/−*^ and KO BMT mice supports the notion that macrophage DJ-1 may play a role in mediating atherogenesis.

**Figure 5:**
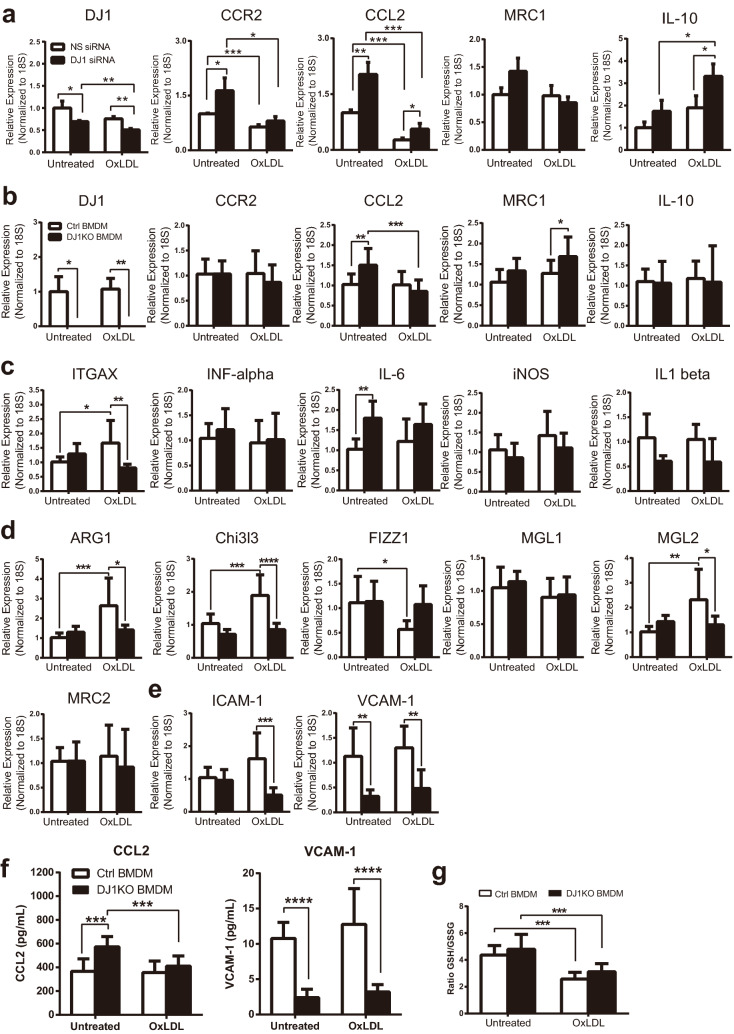
Potential cell-autonomous roles of Dj1 in macrophages. (**a**) mRNA expression of *Dj1*, *Ccr2*, *Ccl2*, *Mrc1*, *Il10* in RAW 264.7 cells transfected with non-specific (NS) and *Dj1* siRNA in response to vehicle (n = 3) or oxLDL (n = 3) (100 μg/mL). Values are normalized to 18S mRNA levels and presented as fold change over untreated NS siRNA. (**b–e**) mRNA expression of (**b**) *Dj1*, *Ccr2*, *Ccl2*, *Mrc1*, *Il10,* as well as **c**) M1 macrophage, (**d**) M2 macrophage*,* and (**e**) transmigration markers in bone-marrow derived macrophages from *Dj1*^*–/–*^*Apoe*^*–/–*^ mice (KO BMDM, *n* = 10) and control *Dj1*^+*/*+^*Apoe*^*–/–*^ mice (control BMDM, *n* = 13) in response to vehicle or oxLDL (100 μg/mL). Values are normalized to 18S mRNA levels and presented as fold change over untreated WT BMDM. (**f**) ELISA analysis of CCL2 and VCAM-1 protein levels in bone-marrow derived macrophages excretion/secretion from *Dj1*^*–/–*^*Apoe*^*–/–*^ mice (KO BMDM, *n* = 9) and *Dj1*^+*/*+^*Apoe*^*–/–*^ mice (control BMDM, *n* = 8) in response to vehicle or oxLDL (100 μg/mL). (**g**). Glutathione levels of bone-marrow derived macrophages from *Dj1*^*−/−*^*Apoe*^*−/−*^ mice (n = 7) and control *Dj1*^+*/*+^*Apoe*^*−/−*^ mice (n = 8). *Dj1* parkinsonism associated deglycase, *Ccr2* C–C Chemokine receptor type 2, *Ccl2* C–C motif chemokine ligand 2, *Mrc1* Mannose receptor C type 1, *Il10* Interleukin 10, *Itgax* Integrin subunit alpha X, *IFN-alpha* Interferons alpha, *IL-6* Interleukin 6, *iNOS *nitric oxide synthases, *Il-1 beta* *Interleukin-1 beta*, *Arg1 *Arginase-1, *Chi3l3* Chitinase-like lectin, *Fizz1* found in inflammatory zone 1, *Mgl1* macrophage galactose-type lectin-1, *Mgl2* macrophage galactose-type lectin-2, *Mrc2* mannose receptor C type 2, *ICAM-1* intercellular adhesion molecule 1, *VCAM-1* vascular cell adhesion Molecule *1.* Data represent mean ± SD. Differences between groups were analyzed for statistical significance by Student unpaired t test and Wilcoxon Rank test. *P < 0.05, **P < 0.01, ***P < 0.001, ****P < 0.0001.

To explore the potential cell-autonomous role of Dj1 in macrophages, mouse macrophage cell line (RAW264.7) was used to assess the expression of pro-inflammatory genes after *Dj1* knockdown. *Dj1* knockdown by siRNA was modest, yet demonstrated increased expression of pro-inflammatory cytokine *Ccl2* and its associated receptor *Ccr2*, with no differences observed in M2-like macrophage marker mannose receptor C-type 1 (MRC1) (Fig. 5a). The addition of oxidized LDL (OxLDL) for 24 h blunted the expression of inflammatory cytokine genes in WT cells as previously shown by others^6,7^. Importantly, OxLDL further blunted the magnitude of cytokine levels in *Dj1* siRNA- compared to non-specific (NS) scramble-transfected macrophages. Moreover, increased expression of anti-inflammatory *Il10* was observed in OxLDL-induced *Dj1* siRNA transfected macrophages (Fig. 5a). Furthermore, BM-derived macrophages (BMDM) from *Dj1*^+*/*+^ *Apoe*^*−/−*^ and *Dj1*^*−/−*^* Apoe*^*−/−*^ mice were treated with PBS control or OxLDL (100 μg/mL) for 24 h followed by gene expression analysis. Similar to RAW264.7 cells with Dj1 knockdown, a significant increase in the expression of pro-inflammatory cytokines *Ccl2* and IL-6 were observed in *Dj1* deficient BMDM compared to controls under basal conditions. However, similar to results from macrophage cell line, *Ccl2* levels were blunted to lower levels in response to oxLDL. Expressions of M1/M2 macrophage markers *Itgax*, *Arg1,* and *Chi3l3* as well as transmigration markers *ICAM-1* and *VCAM-1* were significantly reduced in BMDM with *Dj1* deficiency compared to littermate control (Fig. 5b–e). Furthermore, similar significant reduction of CCL2 and VCAM-1 levels observed in *Dj1* deficient BMDM as assessed by ELISA (Fig. 5f). We next assessed for oxidative stress in BMDM given the role of Dj1 in serving as an anti-oxidant and found no significant differences between *Dj1* deficient BMDM and control group in glutathione levels treated with PBS or OxLDL (Fig. 5g). There were no differences in gene expressions of androgen receptor, or markers of proliferation, or efferocytosis in *Dj1* deficient BMDM compared to controls basally or in response to oxLDL. Interestingly, there was a decrease in *Bcl2* levels both basally and in response to oxLDL in Dj1-deficeint BMDM (Supplementary Figure S3).

### Elevated DJ-1 in subjects with ischemic heart disease

**Figure 6:**
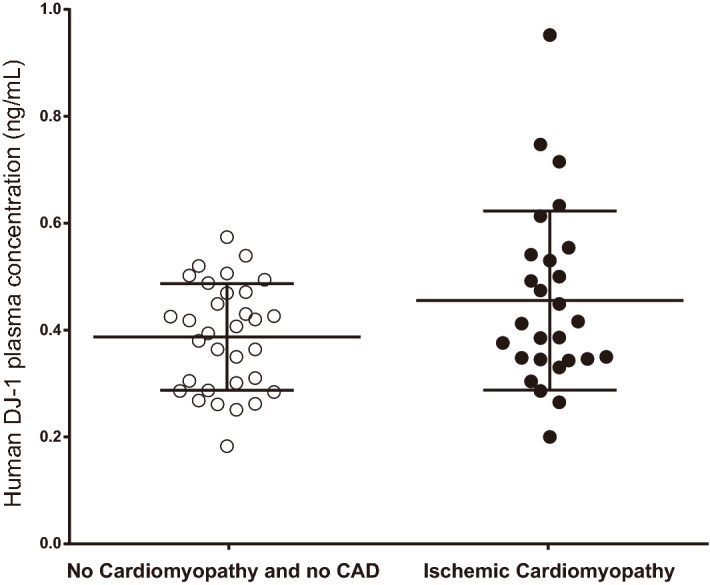
Elevated DJ-1 in subjects with ischemic heart disease. Plasma levels of human DJ-1 from individuals with ischemic cardiomyopathy (n = 27) or no coronary artery disease (CAD) (n = 32). Data represent mean ± SD. Differences between groups were analyzed for statistical significance by Wilcoxon Rank t test.

To assess whether DJ-1 was also affected in humans with atherosclerosis, plasma was collected from subjects with or without ischemic heart disease. Clinical parameters (age, sex, Ejection fraction %, LDL-cholesterol, Total cholesterol) are shown in Table 1. Interestingly, there was a near-significant increase of plasma DJ-1 levels from individuals with ischemic heart disease compared to those without (Fig. 6), similar to our mouse model of atherosclerosis (Fig. 1a). These results further support the pro-atherogenic role of DJ-1 in the regulation of cardiovascular disease.

**Table 1:** Patient characteristics.

	No coronary artery disease	Ischemic cardiomyopathy	P value
Sex (#)	Male (32/32)	Male (27/27)	
Age (years)	56.38 ± 2.33	62.15 ± 1.76	0.0487*
Ejection fraction %	58.20 ± 1.08	36.82 ± 1.60	< 0.0001***
LDL-C (mmol/L)	2.69 ± 0.14	2.30 ± 0.17	0.0966
Total Cholesterol (mmol/L)	4.08 ± 0.15	3.80 ± 0.18	0.2430

## Discussion

In this study, we investigated the role of *Dj1* in the pathogenesis of atherosclerosis. Using mice with *Dj1* deletion, we show that *Dj1*-deficiency attenuates atherosclerosis development and this is associated with a reduction in plaque macrophages. To test whether deletion of *Dj1* in leukocytes had a causal role in attenuating atherosclerosis, we performed bone marrow transplant experiments. We show that irradiated *Apoe*^*-/*^ mice that received bone marrow cells from *Dj1*-deficient mice similarly presented with a reduction in atherosclerosis. Moreover, *Dj1*-deficiency in macrophages enhanced anti-inflammatory activation with M2-like polarization and attenuated pro-inflammatory responses in the presence of oxidized LDL. Together, these data show that *Dj1* promotes atherosclerosis at least in part through regulating macrophage inflammatory responses.

To our knowledge, this is the first study to explore the essential in vivo role of global *Dj1* in a mouse model of atherosclerosis. However, the roles of *Dj1* in disorders of the vasculature have been explored in other models. For example, vascular smooth muscle cells of *Dj1*-deficient mice exhibited greater neointimal formation in response to carotid artery ligation^8^, suggesting a protective role of *Dj1* in vascular remodelling. Furthermore, knockout of *Dj1* aggravated hypoxic pulmonary arterial hypertension in rats^9^. In a model of acetylcholine-induced endothelium-dependent relaxation in the aorta, there was a significant impairment in *Dj1*-deficient mice^10^. Together these results highlight the complex tissue- and context-specific role of DJ-1 in models of vascular pathology.

Immune mechanisms are believed to interact with the adverse metabolic milieu to initiate and propagate atherosclerotic lesions in the intima of arteries^11^. In fact, atherosclerosis is now considered a non-resolving chronic inflammatory condition in which the macrophage is the central player. Thus, the robust attenuation of macrophages within the atherosclerotic plaques of our *Dj1*-deficient mice provides further evidence for the strong link between atherosclerosis and inflammation. Indeed, there is emerging evidence that DJ-1 can modulate immune signaling pathways. For instance, downregulation of *Dj1* in microglial cells has been found to induce their secretion of pro-inflammatory cytokines such as Il-1β and Il-6 and to elevate intracellular ROS^12^. The involvement of DJ-1 in stromal cell-derived factor (SDF)-1 induced CD3^+^ T cell migration has also been reported^13^. Expression of chemokine receptor type 4 (CXCR4) was found to be elevated in CD3^+^ T cells from *Dj1*-deficient mice, suggesting that DJ-1 acts as an inhibitor of inflammatory cell recruitment. On the contrary, *Dj1* knockdown in RAW264.7 cells resulted in reduced expression of pro-inflammatory cytokines, IL-1β, IL-6 and MCP-1, in response to LPS^14^. Our data similarly suggests that *Dj1* knockdown in RAW264.7 cells results in blunted inflammatory response to oxLDL compared to scramble knockdown.

Macrophages become activated as they infiltrate into target tissues and are exposed to stimuli, expressing a highly pro-inflammatory M1-like activation markers. Alternatively, they can acquire an anti-inflammatory phagocytic M2-like activation phenotype. Our results indicate that *Dj1* deficiency in macrophages led to acquiring an anti-inflammatory M2-like state in response to oxLDL. This suggests that *Dj1*-deficiency skews macrophages to a unique activation state that can be characterized by enhanced anti-inflammatory response.

Elevated levels of ROS have been implicated in endothelial dysfunction and inflammation that can exacerbate atherosclerosis^15^. However, the precise mechanisms by which ROS modulate the inflammatory response in atherosclerosis is not clear. DJ-1 has been shown to regulate oxidative stress by directly quenching ROS upon oxidative modification of a conserved cysteine residue^16^ or by stabilizing the master regulator of antioxidant transcription, nuclear factor erythroid-related factor 2 (NRF2)^17^. We previously demonstrated that the protection from diabetes and obesity conferred by *Dj1*-deficiency was found to be mediated by paradoxical elevated levels of ROS which induced metabolic reprogramming within skeletal muscle^4^. Induction of Warburg-like aerobic glycolysis resulted in fuel wasting upon high fat diet feeding as a mechanism leading to protection against obesity and diabetes, through increased energy expenditure.

Circulating DJ-1 has been shown to be induced in models of acute inflammation. Interestingly, we show here a near-significant increase of plasma DJ-1 levels from individuals with ischemic heart disease compared to those without. This group was also more aged, a strong risk factor for cardiovascular disease. Serum DJ-1 levels also increased in atherosclerosis-prone mice in response to atherogenic diet. This suggests that DJ-1 can potentially be a putative biomarker and a therapeutic target for atherosclerosis.

In summary, our data show that loss of *Dj1*, likely in haematopoietic compartment, results in reduced atherosclerosis. The atheroprotection in Dj1-deficient mice may at least in part be related to reduced macrophage inflammatory responses and skewing macrophage activation to an anti-inflammatory state. Limitation of our current study include potential involvement of other cell types including other immune cells such as T cells and endothelial cells which can be regulated by Dj1 in atherogenesis and progression. Future work on mechanistic insight on Dj1 function may further provide the basis for a novel therapeutic approach for the treatment of atherosclerotic cardiovascular disease.

## Methods

### Generation of Dj1/Apoe-null mice

All experiments using mice were approved by the Animal Care Committee at the University Health Network (Toronto, Ontario) and were performed in accordance with ARRIVE relevant guidelines and regulations. *Dj1* knockout mice (*Dj1*^*−/−*^) (generously donated by Dr. Tak W. Mak) were bred to Apolipoprotein E-deficient (*Apoe*^*−/*^ mice (Jackson Laboratory, Stock Number: 002052) to generate *Dj1*^*−/−*^* Apoe*^*−/*^mice. *Dj1*^+*/*+^ *Apoe*^*-/*^littermates served as controls. Only male mice were used for experiments. Mice were fed an atherogenic diet containing 0.2% cholesterol (TD88137, Harlan Laboratories) for 21 weeks, starting at 6 weeks of age. To assess plasma levels of DJ-1 following atherogenic diet, a cohort of *Apoe*^*−/−*^ mice were fed either a standard rodent chow or atherogenic diet for 16 weeks starting at 6 weeks of age.

### Atherosclerotic plaque quantification

Atherosclerotic lesion area was quantified in the descending aorta as previously described^18^.

### Analysis of serum parameters

Mice were fasted for 16 h overnight followed by blood collection. Serum levels of mouse insulin, TNFα, IL-6, CCL2, resistin, and PAI-1 were measured using a mouse serum adipokine kit (Millipore) and the Luminex 100 Instrumental System according to the manufacture’s protocol. Total serum cholesterol and triglycerides, and LDL- and HDL-cholesterol were measured at the Pathology Phenogenomics Core (The Centre for Phenogenomics, Toronto, Ontario). Serum levels of DJ-1 were measured using a mouse DJ-1(PARK7) ELISA kit (Cusabio, Houston, TX), as per the manufacturer’s instructions. Serum H_2_O_2_ levels were determined using the Amplex Red hydrogen peroxide assay kit (Invitrogen) according to the manufacturer’s protocol. Circulating and BMDM levels of GSH to GSSG ratio were determined using a GSH/GSSG ratio detection assay kit (Abcam) as per the manufacturer’s protocol.

### Histology and immunohistochemistry

Sagittal aortic arch sections were stained with haematoxylin and eosin (H&E) and lesion area at the lesser curvature was measured as previously described^18^. Some samples were lost in processing of slides. Immunohistochemical analysis was performed on aortic arch sections using anti-Mac-3 (1:200, M3/84 clone, BD Pharmingen) and anti-α-SMA antibody (1:500, A2547, Sigma-Aldrich) as previously described^18^. Positively stained regions and cells were expressed as percentage of plaque area and total plaque cells, respectively.

### In vivo metabolic analyses

Intraperitoneal glucose (1 g/kg) and insulin (0.75 U/kg) tolerance tests were performed on overnight and 4 h fasted mice, respectively, as previously described^4^. Fasting blood glucose levels were measured after an overnight fast from tail vein using a glucometer (Freestyle Lite).

### Energy homeostasis assessments

To measure energy expenditure, mice were housed individually in a Comprehensive Laboratory Animal Monitoring System (Columbus Instruments) with free access to food and water as previously described^19^. Following 24 h of acclimatization to the apparatus, data for 24 h measurements were collected as previously explained^19^. Respiratory exchange ratio was calculated as VCO_2_/VO_2_. Physical activity was determined by infra-red beam breaks in the x- and z-axes during one measurement interval as previously described^19^.

### Quantitative RT-PCR

Total RNA from bone-marrow derived macrophages and RAW 264.7 cells were isolated using Trizol reagent (Invitrogen) as previously described^18^. RNA was reverse-transcribed with random primers using M-MLV enzyme (Invitrogen) as previously reported^18^. Quantitative RT-PCR (qRT-PCR) was performed with specific primers and SYBR Green master mix using a 7900HT Fast-Real-Time PCR System (Applied Biosystems) as previously described^18,20^. mRNA abundance was evaluated using a standard curve method specific for each primer analyzed. The relative mRNA abundance of each gene was normalized to the expression levels of 18S. Primer sequences are listed in Supplementary Table S1.

### Cell culture

RAW 264.7 murine macrophage cells (TIB-71, American Type Culture Collection) were cultured in DMEM (4.5 g/L d-glucose) supplemented with 10% (vol/vol) FBS, 100 units/mL penicillin and streptomycin (Gibco) at 37 °C with 5% CO_2_ in humidified air as previously stated in^21^.

### siRNA transfection

RAW 264.7 cells in growth medium were transfected with 50 nM scramble or Silencer Select Dj1 siRNA (s81228, ThermoFischer Scientific) using Lipofectamine RNAiMAX reagent (Invitrogen) according to the manufacturer’s protocol. Cells were transfected for 24 h followed by addition of oxidized low-density lipoprotein (oxLDL; 100 μg/mL) for another 24 h.

### Bone marrow-derived macrophages

Bone marrow cells were harvested from femurs of control and *Dj1*-deficient mice and differentiated into macrophages for 7 days in differentiation media (DMEM with 10% (v/v) FBS, 20% (v/v) L929 conditioned media and 1% (v/v) penicillin and streptomycin) to generate bone marrow-derived macrophages as previously reported^22^. Cells were then treated with PBS or oxLDL (100 μg/mL; Kalen Biomedical) for another 24 h before harvesting.

### Protein analysis with ELISA

CCL2 (Mouse CCL2/JE/Mcp-1 Quantikine ELISA Kit, R&D Systems) and VCMA-1 (Mouse VCAM-1/CD106 Quantikine ELISA Kit, R&D Systems) levels from BMDM treated with PBS or oxLDL were analyzed via ELISA according to manufacturers’ instructions.

### Flow cytometry

Antibodies used for flow cytometry are provided in Table 2. Data were acquired on an LSRII flow cytometer (BD Biosciences) and analyzed with FlowJo v8.8.6 (Tree Star, Inc.) as previously described^22^.

**Table 2:** List of antibodies used for FACS analysis.

Antigen	Company	Clone
Ly6C	BD	AL-21
CD115	eBiosciences	AF598
CD45.2	eBiosciences	104
B220	BD	RA3-6B2
CD3e	BD	17A2
Ly6G	BD	1A8

### Human samples

Serum from patients with ischemic cardiomyopathy or control group without coronary artery disease but with valvular heart disease were obtained for analysis of serum DJ-1. Patients were recruited from the Division of Cardiology at the McGill University Health Center. The research protocol was reviewed and approved by the Research Ethics Board of the McGill University Health Center (MUHC-BMB-06-012). All research was performed in accordance with relevant guidelines and regulations; all subjects gave written informed consent to participate as previously reported^23^.

### Statistics

Data are presented as mean ± SD. Statistical tests were performed using GraphPad Prism version 5 (GraphPad Software, La Jolla, CA, USA). Data were tested for normal distribution using D'Agostino & Pearson and Shapiro–Wilk normality tests. Data with normal distribution were then subjected to unpaired parametric student t test. Non-normally distributed data were analyzed using unpaired non-parametric t test of Wilcoxon rank (Mann–Whitney). Data analyzed using multiple t tests followed a multiple test correlation. Statistical significance was defined as a P value < 0.05.

## Supplementary Information

Supplementary information.
