# Supplementary material for: Dj1 deficiency protects against atherosclerosis with anti-inflammatory response in macrophages
Source: Sci Rep. 2021 Feb 25;11:4723. doi: 10.1038/s41598-021-84063-6 (PMC7907332; doi:10.1038/s41598-021-84063-6)
Supplement: Supplementary file 1 — Supplementary information. [file 41598_2021_84063_MOESM1_ESM.docx]

**Dj1-deficiency protects against atherosclerosis with anti-inflammatory response in macrophages**

Tharini Sivasubramaniyam, Jiaqi Yang, Henry S. Cheng, Alexandra Zyla, Angela Li, Rickvinder Besla, Idit Dotan, Xavier S. Revelo, Sally Y. Shi, Helen Le, Stephanie A. Schroer, David W. Dodington, Yoo Jin Park, Min Jeong Kim, Daniella Febbraro, Isabelle Ruel, Jacques Genest, Raymond H. Kim, Tak W. Mak, Daniel A. Winer, Clinton S. Robbins, and Minna Woo

Supplementary Table S1: Primer sequences for RT-qPCR analysis (mouse).

| **Gene** | **Forward (5'-3')** | **Reverse (5'-3')** |
| --- | --- | --- |
| *Dj1* | ATCTGAGTCGCCTATGGTGAAG | ACCTACTTCGTGAGCCAACAG |
| *Ccr2* | ATCCACGGCATACTATCAACATC | CAAGGCTCACCATCATCGTAG |
| *Ccl2* | GCCCTAAGGTCTTCAGCACCTT | TGCTTGAGGTGGTTGTGGAA |
| *Mrc1* | CTGGACAAACTGGTCCACCT | TCCCCTTCTCTCCCTTTTGT |
| *Il10* | GCTGGACAACATACTGCTAACC | CCCAAGTAACCCTTAAAGTCCTG |
| *18S* | AGTCCCTGCCCTTTGTACACA | CGATCCGAGGGCCTCACTA |
| *F4/80* | TGACTCACCTTGTGGTCCTAA | CTTCCCAGAATCCAGTCTTTCC |
| *Cd68* | TGTCTGATCTTGCTAGGACCG | GAGAGTAACGGCCTTTTTGTGA |
| *Itgax* | CTGGATAGCCTTTCTTCTGCTG | GCACACTGTGTCCGAACTCA |
| *Infa* | CTGTGAAGGGAATGGGTGTT | TTGGACCCTGAGCCATAATC |
| *Il6* | CTCTGGGAAATCGTGGAAATG | AAGTGCATCATCGTTGTTCATACA |
| *iNOS* | GTTCTCAGCCCAACAATACAAGA | GTGGACGGGTCGATGTCAC |
| *Il1b* | GAAATGCCACCTTTTGACAGTG | TGGATGCTCTCATCAGGACAG |
| *Arg1* | CTCCAAGCCAAAGTCCTTAGAG | GGAGCTGTCATTAGGGACATCA |
| *Chi3l3* | CAGGTCTGGCAATTCTTCTGAA | GTCTTGCTCATGTGTGTAAGTGA |
| *Fizz1* | CCAATCCAGCTAACTATCCCTCC | ACCCAGTAGCAGTCATCCCA |
| *Mgl1* | CACCATGATATACGAAAACCTCCAGAACTC | CTAGCTCTCCTTGGCCAGC |
| *Mgl2* | CAATGTGCTTAGCTGGATGGG | CCATGCCAGTTATCCGGCTG |
| *Mrc2* | TCTCCCGGAACCGACTCTTC | AACTGGTCCCCTAGTGTACGA |
| *Ccr5* | TTTTCAAGGGTCAGTTCCGAC | GGAAGACCATCATGTTACCCAC |
| *Ccr7* | TGTACGAGTCGGTGTGCTTC | GGTAGGTATCCGTCATGGTCTTG |
| *Ccl3* | TTCTCTGTACCATGACACTCTGC | CGTGGAATCTTCCGGCTGTAG |
| *Ccl4* | TTCCTGCTGTTTCTCTTACACCT | CTGTCTGCCTCTTTTGGTCAG |
| *Ccl5* | GCTGCTTTGCCTACCTCTCC | TCGAGTGACAAACACGACTGC |
| *Ccl7* | GCTGCTTTCAGCATCCAAGTG | CCAGGGACACCGACTACTG |
| *Cxcl4* | CAGTCCTGAGCTGCTGCTTCT | TCCAGGCTGGTGATGTGCTTA |
| *ICAM-1* | GTGATGCTCAGGTATCCATCCA | CACAGTTCTCAAAGCACAGCG |
| *VCAM-1* | AGTTGGGGATTCGGTTGTTCT | CCCCTCATTCCTTACCACCC |
| *Ar* | TCCAAGACCTATCGAGGAGCG | GTGGGCTTGAGGAGAACCAT |
| *CyclinD* | GCGTACCCTGACACCAATCTC | ACTTGAAGTAAGATACGGAGGGC |
| *Pcna* | TTTGAGGCACGCCTGATCC | GGAGACGTGAGACGAGTCCAT |
| *p53* | CCCCTGTCATCTTTTGTCCCT | AGCTGGCAGAATAGCTTATTGAG |
| *p21* | CCTGGTGATGTCCGACCTG | CCATGAGCGCATCGCAATC |
| *Bax* | TGAAGACAGGGGCCTTTTTG | AATTCGCCGGAGACACTCG |
| *Bcl2* | GAGAGCGTCAACAGGGAGATG | CCAGCCTCCGTTATCCTGGA |
| *BclXL* | GACAAGGAGATGCAGGTATTGG | TCCCGTAGAGATCCACAAAAGT |
| *Abca1* | AAAACCGCAGACATCCTTCAG | CATACCGAAACTCGTTCACCC |
| *Abcg1* | ATCTGAGGGATCTGGGTCTGA | CCTGATGCCACTTCCATGA |
| *Srb1* | TCCCTTCGTGCATTT TCTCA | GTTCATCCCAACAAACAGGC |

**Supplementary Figure S1**


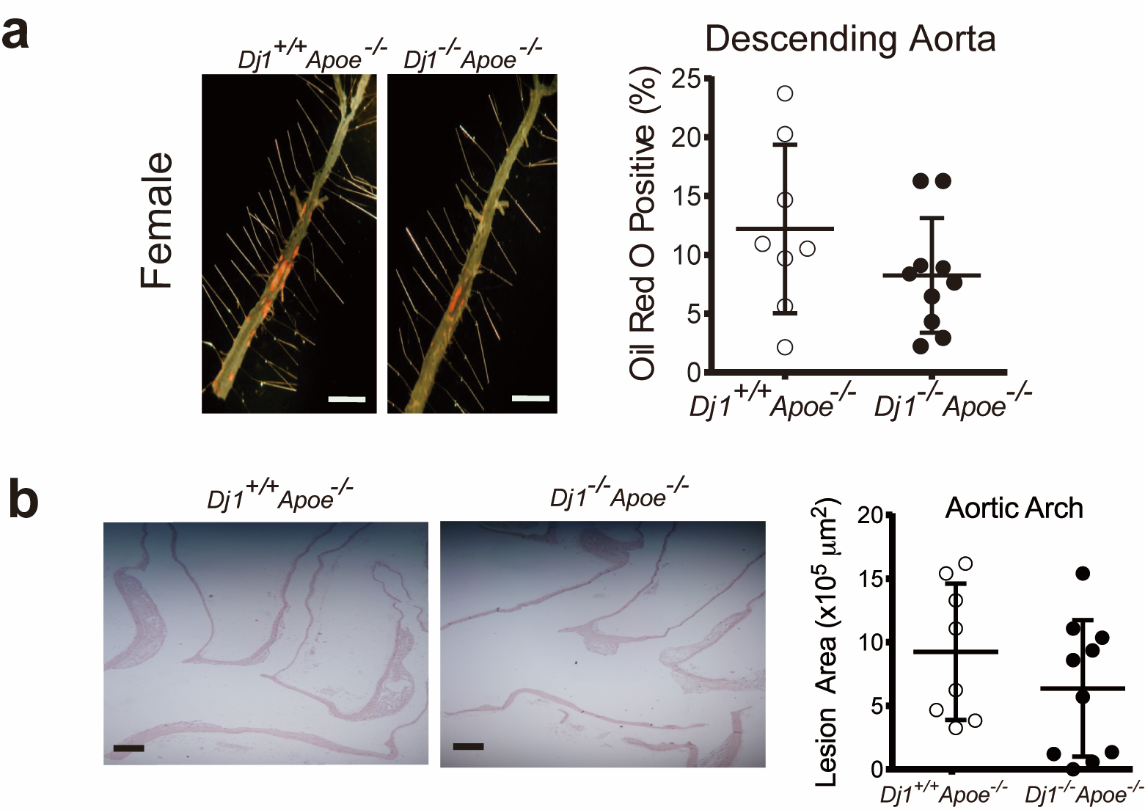


**Supplementary Figure S1: Dj1 deficiency leads to similar atherogenesis in females compared to littermates. a)** Representative photographs of *en face* Oil-red-O (ORO) staining and quantification of atherosclerotic plaque area in descending aortas of female *Dj1^-/-^Apoe^-/-^* (n=10) and control *Dj1^+/+^Apoe^-/-^* (n=8) mice. Scale bar: 5 mm. P=0.18**. b)**. Representative images of the lesser curvature of longitudinal aortic arch from female *Dj1^-/-^Apoe^-/-^* (n=10) and control *Dj1^+/+^Apoe^-/-^* (n=8) mice stained with H&E and quantification of lesion size, respectively. Scale bar: 200 μm. P=0.27**.** Data represent mean ± SD. Differences between groups were analyzed for statistical significance by Student unpaired t test.

**Supplementary Figure S2**


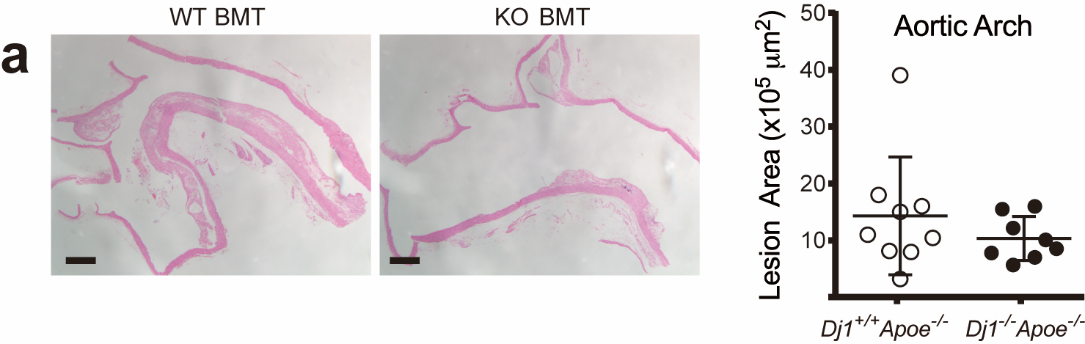


**Supplementary Figure S2: Atherosclerotic plaque in arch of BMT model.** **a)** Representative images of the lesser curvature of longitudinal aortic arch sections from KO BMT mice (n=8) and WT BMT mice (n=9) stained with H&E and quantification of lesion size at the lesser curvature. Scale bar:200 μm. Data represent mean ± SD. Differences between groups were analyzed for statistical significance by Student unpaired t test and Wilcoxon Rank test.

**Supplementary Figure S3**


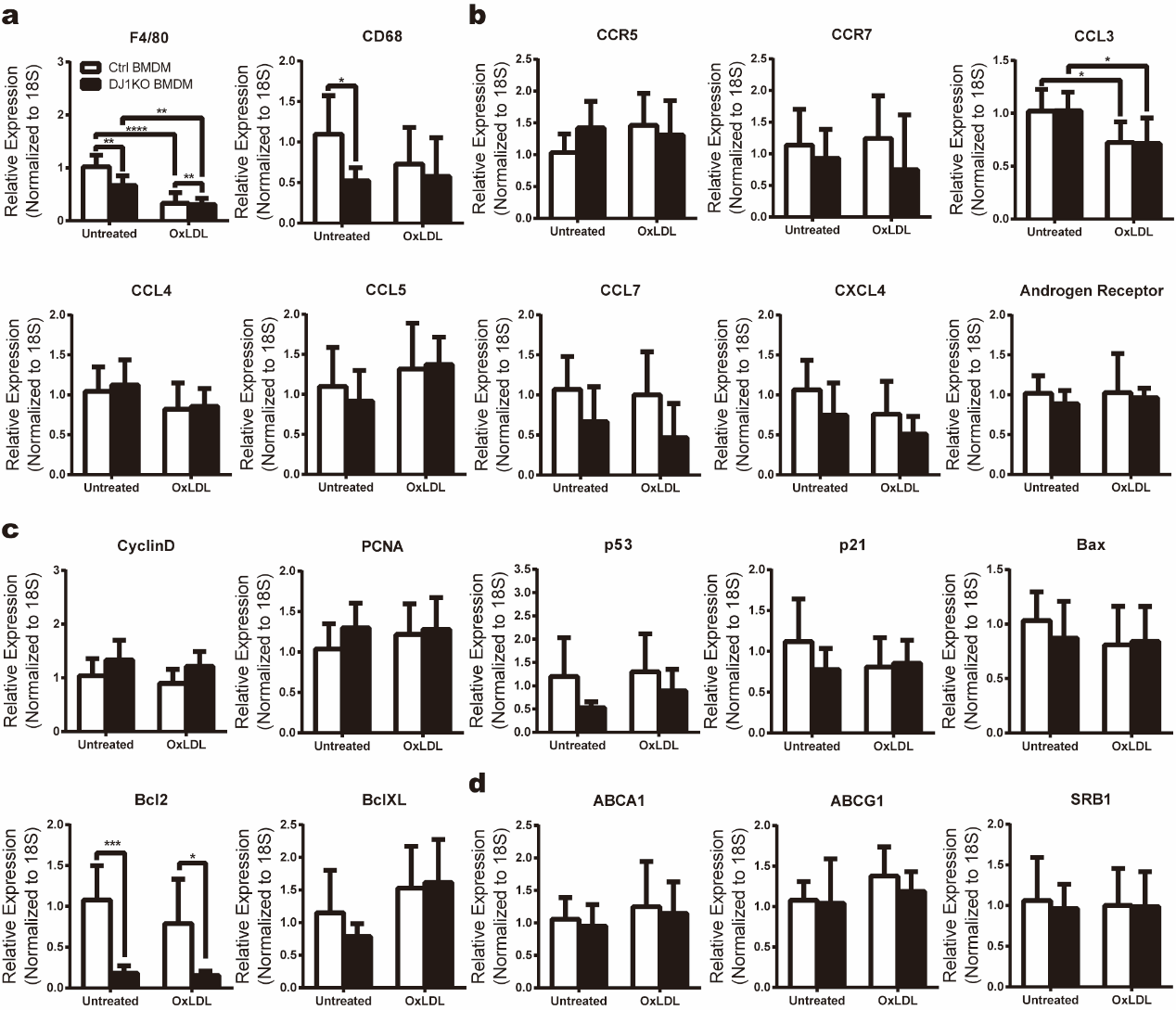


**Supplementary Figure S3: Gene expressions of macrophage, chemokine and receptors, proliferation and apoptosis, as well as efferocytosis markers in BMDM with Dj1 deficiency.** mRNA expressions of **a)** M0 macrophage markers, **b)** chemokine and receptors, **c)** proliferation and apoptosis, and **d)** efferocytosis markers in bone-marrow derived macrophages from *Dj1*^–/–^Apoe^–/–^ mice (KO BMDM, n=7) and control *Dj1*^+/+^Apoe^–/–^mice (control BMDM, n=10) in response to vehicle or oxLDL (100μg/mL) is assessed by RT-qPCR. Values are normalized to 18S mRNA levels and presented as fold change over untreated control BMDM. Data represent mean ± SD. Differences between groups were analyzed for statistical significance by Student unpaired t test and Wilcoxon Rank test. *P< 0.05, **P< 0.01, ***P<0.001, ****P<0.001. *Pcna:* Proliferating cell nuclear antigen*;* *Bax*: Bcl-2-like protein 4; *Bcl2* B-cell lymphoma 2; *BclXL:* B-cell lymphoma-extra large; *Abca1*: ATP-binding cassette transporter; *Abcg1*: ATP binding cassette subfamily G member 1; *Srb1:* Scavenger receptor class B type 1.
